# Thoracic splenosis: Precision medicine can prevent thoracic surgery

**DOI:** 10.1002/rcr2.70067

**Published:** 2024-11-21

**Authors:** Nathan Harb, Julia Fattore, Mayuran Saththianathan, Stephen Parsons

**Affiliations:** ^1^ Department of Thoracic Medicine St. Vincent's Hospital Sydney New South Wales Australia; ^2^ School of Clinical Medicine St Vincent's Healthcare Clinical Campus, Faculty of Medicine and Health, UNSW Sydney Sydney New South Wales Australia; ^3^ Macquarie Respiratory and Sleep Macquarie University Hospital Sydney New South Wales Australia; ^4^ Department of Nuclear Medicine Western Sydney Local Health District Sydney New South Wales Australia; ^5^ Respiratory and Sleep Medicine South Western Sydney Local Health District Sydney New South Wales Australia

**Keywords:** lung mass, splenectomy, thoracic splenosis

## Abstract

Thoracic splenosis is a rare condition referring to the auto‐transplantation of splenic tissue into the thoracic cavity following splenic trauma. We present a case of thoracic splenosis in a 62‐year‐old man who at the age of 17 suffered three gunshot wounds to the posterior thorax and abdomen, requiring a splenectomy and intercostal catheter insertion. In 2007, he underwent a thoracotomy and biopsy of a left sided pulmonary mass which was complicated by a haemothorax requiring an emergent return to theatre and rib resection to achieve haemostasis. The biopsy revealed granulation tissue. In 2023, Technetium‐99m (Tc‐99m) heat‐damaged erythrocyte scintigraphy confirmed the diagnosis of thoracic splenosis. This case highlights the importance of recognizing this uncommon condition to prevent unnecessary investigation, as well as the use of Tc‐99m heat‐damaged erythrocyte scintigraphy to confirm the diagnosis.

## Graphical abstract

We present a case of thoracic splenosis in a 62‐year‐old man. This case highlights the importance of recognizing this uncommon condition to prevent unnecessary interventions and complications.

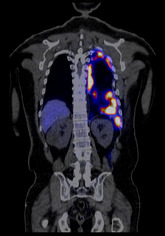

## INTRODUCTION

Thoracic splenosis is a rare, benign condition characterized by the presence of ectopic splenic tissue within the thoracic cavity, often following splenic trauma. Splenosis can occur in either the peritoneal or, more rarely, the pleural cavity, with the latter occurring with simultaneous diaphragmatic rupture. The diagnosis of thoracic splenosis is difficult owing to its rarity and its radiological resemblance to malignancy. As such, accurate diagnosis is essential to prevent unnecessary invasive investigation.

## CASE REPORT

A 62‐year‐old building contractor of Lebanese descent was referred to a suburban Respiratory Clinic in New South Wales, Australia in 2023 for further investigation of multiple lobulated pleural masses in the left hemithorax resembling possible mesothelioma. These pulmonary changes were noted during a recent hospital admission for community acquired pneumonia.

He reported an episodic productive cough, but denied dyspnoea, chest pain, haemoptysis or constitutional symptoms. His medical background was significant for chronic back pain, occupational asbestos exposure and previous cigarette smoking with a 20 pack‐year history. He denied a history of childhood lung disease. He did, however, report that he required a splenectomy and an intercostal catheter insertion after being shot twice in the thorax and once in the abdomen during combat in the Lebanese civil war at the age of 17.

In 2007, he had been admitted to his local hospital for investigation of chest pain and was found to have multiple lobulated pulmonary masses on computed tomography. At that time, he underwent a thoracotomy and surgical lung biopsy which was complicated by an intercostal bleed and haemothorax requiring an emergent repeat procedure and rib resection for haemostasis. The biopsies revealed granulation tissue.

**FIGURE 1:**
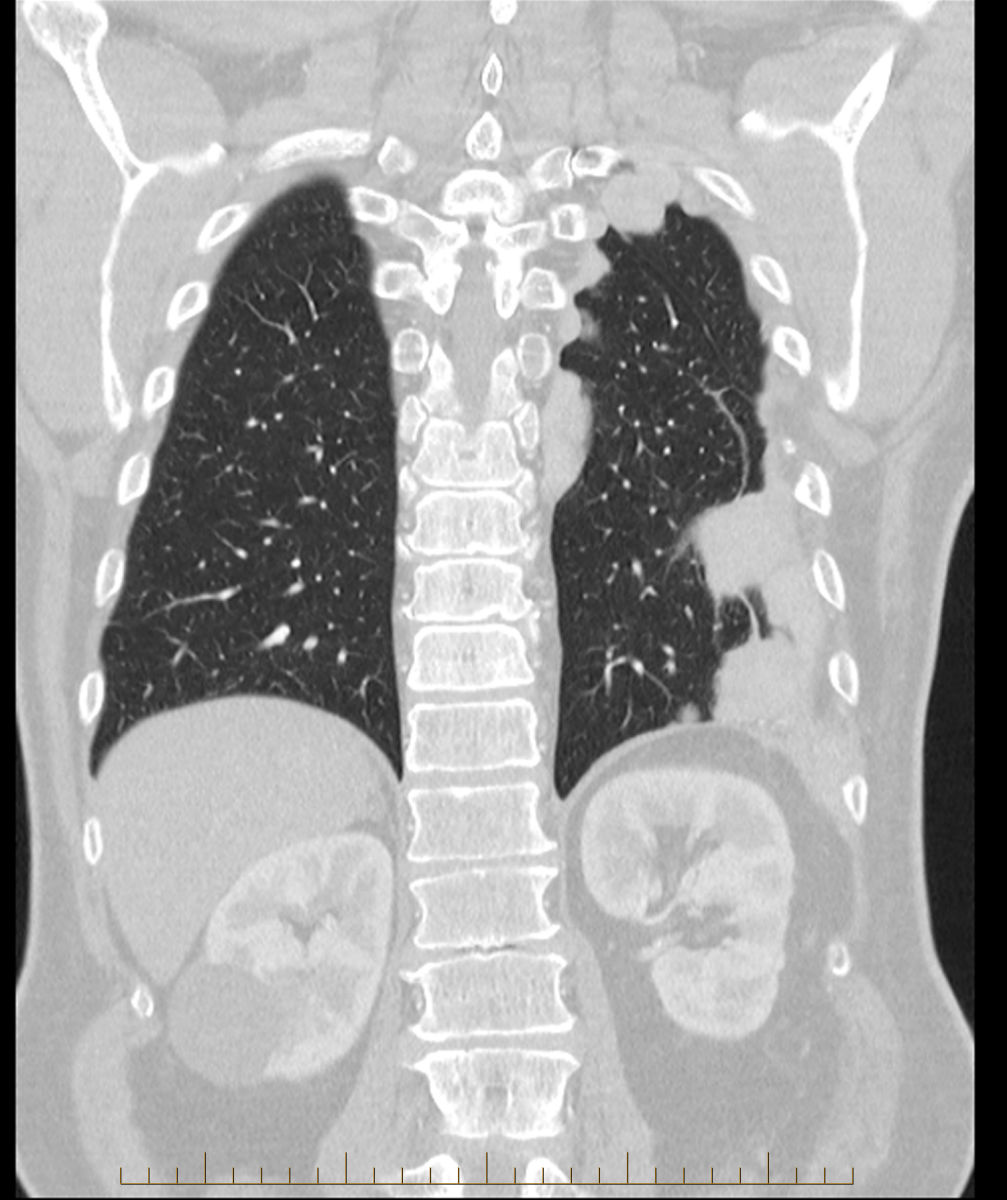
A coronal slice from a computed tomography scan of the chest showing lobulated pleural based masses with some round atelectasis.

On examination, oxygen saturations were 98% on room air with vesicular breath sounds on auscultation. Examination of the anterior abdomen revealed a midline laparotomy scar, a left anterior thorax intercostal drain scar and a left subphrenic drain site scar. The posterior thorax revealed a left thoracotomy scar and a drain site wound. Spirometric indices were normal. Recent computed tomography of the chest demonstrated multiple lobulated pleural‐based masses with volume loss in the left lower lobe of the lung with a bullet still present in the left upper lobe. These masses were unchanged in appearance compared to his prior imaging in 2007 (Figure 1).

*A diagnostic test was performed. What is the name of the diagnostic test?*

**FIGURE 2:**
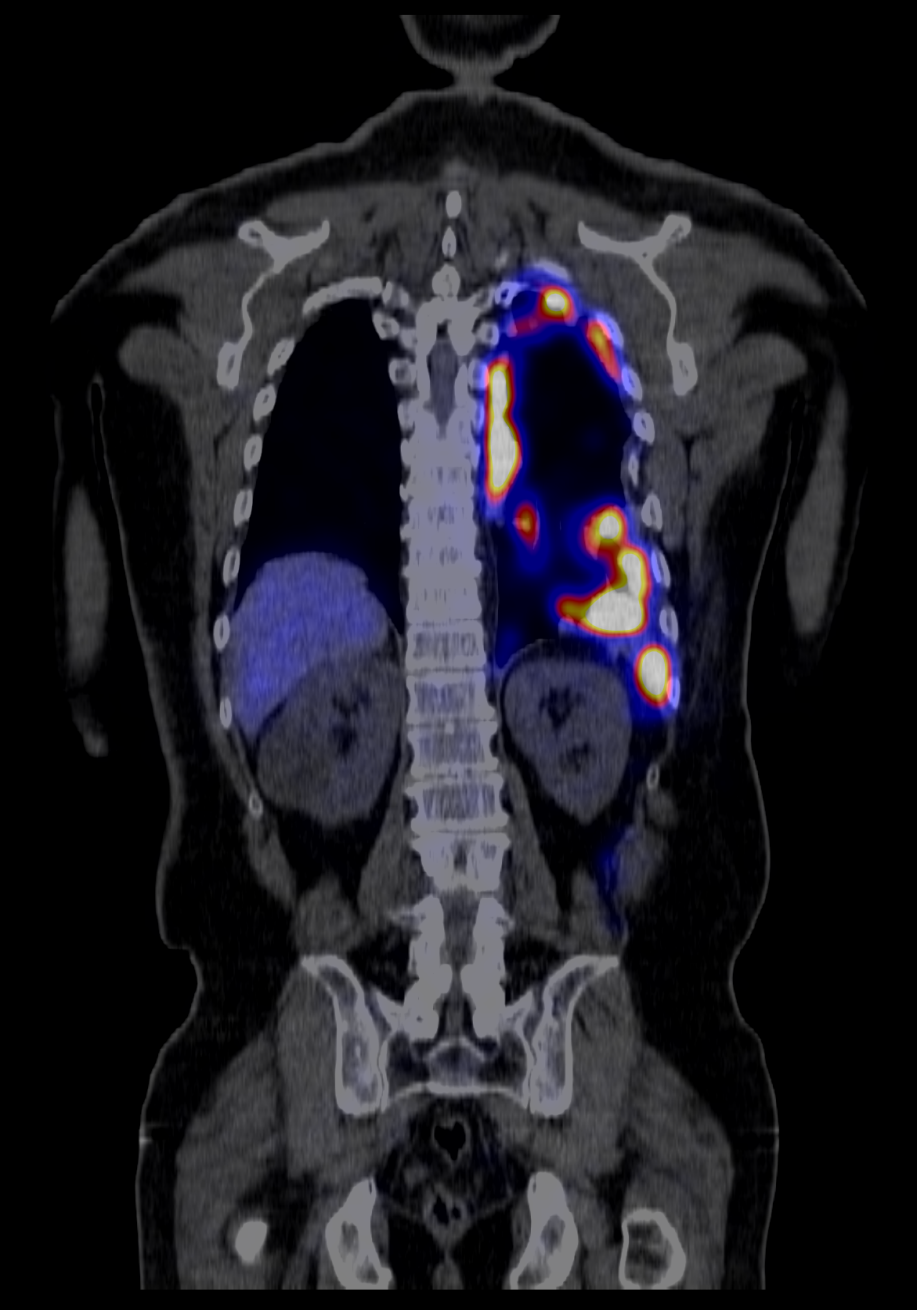
A coronal slice from the Tc‐99m heat‐damaged erythrocyte scintigraphy scan showing intense uptake in the left hemithorax.

Technetium‐99m (Tc‐99m) heat‐damaged erythrocyte scintigraphy demonstrated radiopharmaceutical uptake in the pleural masses, most pronounced posteriorly along the costal pleura as well as the diaphragmatic pleura, in keeping with a diagnosis of thoracic splenosis (Figure 2). There was further uptake in the left hilum, left oblique fissure and within the abdomen. He was counselled regarding his condition and discharged from the clinic for ongoing review by his primary care physician.

## DISCUSSION

This is a classic case of thoracic splenosis which highlights the importance of recognizing the condition to prevent unnecessary investigation, namely biopsy‐related complications and surgical interventions given a recognized increased risk of bleeding.^1^ This case also supports the use of Tc‐99m heat‐damaged erythrocyte scintigraphy to confirm the diagnosis.

Thoracic splenosis was first described by Shaw and Shafi in 1937 and is a rare condition with approximately 76% of cases occurring in males. While splenosis is estimated to occur in approximately 67% of splenic rupture cases, thoracic splenosis is estimated to occur in approximately 18% of cases of splenic rupture.^2^ It is likely under‐reported given it is often asymptomatic and found incidentally. Historically, gunshot wounds were the most common cause of splenic rupture, accounting for approximately 50% of cases in the literature, however motor vehicle accidents account for 36% of thoracic splenosis cases, and this proportion is rising over time.^2^

Thoracic splenosis is asymptomatic in 60% of cases and rarely it can present with haemoptysis and chest pain.^3^ Radiologically it can present with numerous nodules of various sizes (0.5–7 cm) most commonly in the left thorax. The preferred imaging modality for diagnosis of thoracic splenosis is Tc‐99m heat‐damaged erythrocyte scintigraphy. This modality is both highly specific and sensitive as Tc‐99m heat damaged erythrocytes are sequestered by reticuloendothelial cells and can be used to identify splenic tissue.^4^, ^5^ It is important to note that this tissue is functional spleen and may increase in size over time, potentially mimicking malignancy.^5^ Biopsy of splenosis usually reveals distorted architecture with variable histology and carries the risk of biopsy‐related complications.^4^

By diagnosing this condition with this nuclear medicine scan, further investigations such as bronchoscopy, biopsy, thoracoscopy and thoracotomy are not required. Given thoracic splenosis lesions are functional spleen and that pleural adhesions are likely to be present, thoracotomy should generally be avoided due to the high risk of bleeding.^2^

In conclusion, thoracic splenosis is a rare condition that classically presents with incidentally found pleural based nodules on a background of splenic rupture or splenectomy. Biopsy‐related complications can be prevented by recognizing the condition, and by requesting a Tc‐99 m heat‐damaged erythrocyte scintigraphy scan. Ongoing education is needed to raise awareness for the diagnosis of thoracic splenosis to prevent unnecessary intervention and complications.

## AUTHOR CONTRIBUTIONS

Dr. Nathan Harb contributed to the design of the work, the acquisition of data, drafting and revising the work, provided final approval of the version to be published, and agreed to be accountable for all aspects of the work. Dr. Julia Fattore contributed to the design of the work, the acquisition of data, drafting and revising the work, provided final approval of the version to be published, and agreed to be accountable for all aspects of the work. Dr. Mayuran Saththianathan contributed to the design of the work, the acquisition of data, drafting and revising the work, provided final approval of the version to be published, and agreed to be accountable for all aspects of the work. Dr. Stephen Parsons contributed to the design of the work, the acquisition of data, drafting and revising the work, provided final approval of the version to be published, and agreed to be accountable for all aspects of the work.

## CONFLICT OF INTEREST STATEMENT

None declared.

## ETHICS STATEMENT

The authors declare that appropriate written informed consent was obtained for the publication of this manuscript and accompanying images.

## Data Availability

Data sharing is not applicable to this article as no new data were created or analyzed in this study.
